# Healing Hearts Together — an emotionally focused intervention for couples after a cardiac event: a randomized controlled trial protocol

**DOI:** 10.3389/fpsyg.2025.1564666

**Published:** 2025-07-22

**Authors:** Heather E. Tulloch, Paul S. Greenman, Jennifer Reed, Stephanie Susinski, Eniko Kasos, Giorgio A. Tasca, Lisa Mielniczuk, George Wells, Louise Y. Sun, Susan M. Johnson

**Affiliations:** ^1^University of Ottawa Heart Institute, Ottawa, ON, Canada; ^2^Department of Medicine and School of Psychology, University of Ottawa, Ottawa, ON, Canada; ^3^Département de Psychoéducation et de Psychologie, Université du Québec en Outaouais, Gatineau, QC, Canada; ^4^Mayo Clinic, Rochester, MN, United States; ^5^Stanford University School of Medicine, Stanford, CA, United States; ^6^International Centre for Excellence in Emotionally Focused Therapy, Ottawa, ON, Canada

**Keywords:** cardiovascular disease, couples, relationship quality, intervention, emotionally focused therapy (EFT), attachment

## Abstract

**Introduction:**

Couple relationships are important for health. Relationship distress is associated with increased incident and prognostic cardiovascular risk, while positive support is linked to heart-healthy behaviors and improved outcomes. This paper describes the study rationale, objectives, design, and methods of the Healing Hearts Together (HHT) randomized controlled trial (RCT).

**Objectives:**

The primary objective is to examine the difference in relationship quality between the 8-week HHT intervention group and usual care (UC) at program completion. Secondary objectives include evaluating the impact of HHT on relationship quality at 6 months, and mental health, quality of life, and cardiovascular risk factors measured at 8 weeks and 6 months post-intervention, as compared to usual care.

**Methods:**

Patients and their partners are recruited within 6 months of a cardiac event, procedure, or hospitalization and randomized 1:1 to HHT or UC. Assessments occur at baseline, 8 weeks, and 6 months follow-up. Analyses are planned as intention-to-treat, with multi-level analyses of covariance (ANCOVA) for the primary outcome: 8-week relationship quality as measured by the Dyadic Adjustment Scale. Secondary objectives will be evaluated using multi-level modeling for repeated measures.

**Anticipated results:**

It is expected that participants randomized to HHT will report higher relationship quality and improved secondary outcomes than will participants in UC.

**Conclusion:**

As the first study to evaluate a relationship-enhancement program for couples with cardiac disease, findings will have important clinical implications regarding the effect of relationship interventions on heart health.

## Background

The quality of couple relationships significantly affects both psychological and physical health, particularly in the context of cardiovascular disease (CVD) (Azizi et al., 2024). High-quality relationships, characterized by satisfaction and minimal hostility, are linked to better health outcomes, whereas strained relationships or a sense of disconnection from close others can lead to adverse outcomes. To illustrate, a meta-analysis involving 35,925 participants reported that loneliness and social isolation increased the risk of coronary artery disease (CAD) by 29% (Valtorta et al., 2016). In contrast, positive support provided by a partner can protect against cardiac-related health issues, including metabolic syndrome and post-operative complications. For example, patients reporting satisfying relationships are 3 times more likely to be alive at 15 years post-bypass surgery (King and Reis, 2012).

Couples’ relationship quality (RQ) may influence heart health both directly and indirectly. Direct pathways include the impact of psychological and physical challenges on the cardiovascular, neuroendocrine, and immune systems. Psychological stress from relationship strain or hostility can activate the sympathetic nervous system, leading to increased blood pressure, heart rate, and cardiac output (Valtorta et al., 2016; Kelli et al., 2019). Supportive relationships, on the other hand, are linked to improved heart rate variability (HRV), a measure of the heart’s ability to respond to stress. Higher HRV is associated with better cardiovascular health (Goodyke et al., 2024) and lower stress hormone levels, reducing the risk of CAD and mortality (Pourmand et al., 2023). Further, couples in which one partner criticizes while the other shuts down emotionally secrete higher levels of cortisol during conflict. Elevated cortisol levels are strong predictors of CAD and cardiac mortality (Shrout et al., 2020).

Indirect pathways in which RQ influences heart health involve health behaviors driven by RQ (Azizi et al., 2024; Khaing et al., 2017; Rapelli et al., 2022; Bertoni et al., 2023). Relationship problems can lead to engagement in risky health behaviors such as smoking and alcohol use (Roberson et al., 2018), whereas better marital adjustment and positive dyadic coping is associated with enhanced medication compliance (Rapelli et al., 2022) and cardiac rehabilitation (CR) (Trevino et al., 1990). Spouses can exert social influence over health behaviors, such as preparing low-sodium meals, modeling healthy behavior (e.g., exercise), or assisting in the management of disease (e.g., medication management) (Ford et al., 2009). However, if this support is in the form of overprotection, for example, it may cause more harm than good (Bertoni et al., 2023). Satisfying relationships are associated with lower rates of mental health problems and better quality of life in cardiac patients, whereas discord can exacerbate anxiety and depression (Bouchard et al., 2021; Bouchard et al., 2023), increasing the risk of cardiac complications and death (Civieri et al., 2024).

Current cardiac interventions often overlook the importance of RQ. Consequently, few studies exist that measure the effect of couples-based interventions in patients with heart disease and their partners. Existing research has focused on traditional approaches such as diet, exercise, and stress management, producing inconsistent results. A recent scoping review (Rapelli et al., 2023) of couple-based psychological interventions for cardiac patients and their partners identified 11 studies (6 RCTs). Many interventions were classified as “partner-assisted” (i.e., partners complete tasks to help the patient), with the remaining studies being “couple-interaction” interventions in which couple adjustment and coping were addressed (Gartner et al., 1988; Lenz and Perkins, 2000; Sher et al., 2014; Stewart et al., 2001). Of the latter studies (3 RCTs), the interventions showed little improvement over control conditions. Gartner et al. (1988) reported increased patient tolerance for emotional distress at 3 months post surgery, but no differences were found at 6 months, and no partner effects were observed. An enhanced version of this intervention did not produce differences between intervention and control groups (Lenz and Perkins, 2000). Lastly, compared to a patient-only education program for cardiac risk reduction, couples receiving education plus counseling on communication skills and relationship issues reported higher levels of physical activity, but no differences were detected on any of the other variables of interest (e.g., body mass index, nutrition, medication compliance) (Sher et al., 2014). As such, there has been a call in the literature for high-quality, contemporary studies of couples facing heart disease that includes enhanced relationship-focused interventions and measures of RQ for the patient and partner to enhance intervention success (Rapelli et al., 2023; Pietromonaco and Collins, 2017; Pietromonaco et al., 2013; Smith, 2022). The inclusion of partners in the care of patients with CVD has the potential to not only enhance patient outcomes, but also to help partners who often share similar cardiovascular risk factors.

With its solid foundation of evidence (Mikulincer and Shaver, 2016; Cassidy and Shaver, 2016) relationship scientists have applied attachment theory to understand the link between RQ and health, as well as to address the role of relationship distress in the health context. Attachment theory specifies that all human beings have an innate need for emotional connections with significant others and, when these ties are threatened (e.g., during a cardiac event), immense distress is triggered and attachment behaviors (e.g., seeking closeness) as well as caregiving behaviors (e.g., protection) are activated in an attempt to gain reassurance and/or emotional homeostasis. Emotionally focused, attachment-based interventions have been applied to help couples solidify a sense of security and connection by articulating and reaching out when feeling vulnerable (e.g., “what would I do if you died?”). These interventions have produced improved relationship satisfaction and health outcomes (e.g., reduce heart rate and cortisol levels) during stressful situations (Greenberg and Watson, 2006; Johnson, 2019; Johnson and Denton, 2002).

In the cardiac context, members of our team created the *Healing Hearts Together* (HHT) program, adapted from the *Hold Me Tight: Conversations for Connection* program (Johnson, 2010). Rooted in attachment theory, HHT emphasizes the need for close emotional bonds between partners and introduces strategies to address the emotional challenges of heart disease to promote healthy coping (Tulloch et al., 2021). Our proof-of-concept data demonstrated clinically significant improvements from pre- to post-intervention on relationship distress, depression, anxiety and quality of life (Tulloch et al., 2021) among patients with heart disease and their partners. However, no lifestyle, physiological (e.g., inflammatory makers, cortisol reactivity), or cardiovascular risk factors or outcomes were measured, and a comparison group was not included. Further study is needed to definitively determine the clinical impact and underlying mechanisms of HHT.

## Study aims and objectives

The primary aim of this RCT is to examine the efficacy of the 8-week HHT program on the relationship quality of patients with CVD and their romantic partners at 8 weeks, in comparison to UC. We expect that those assigned to the HHT condition will have greater improvements in RQ scores (as measured by the dyadic adjustment scale) than will those in UC. Secondary aims include measuring the impact of the program on relationship quality at 6 months and secondary outcomes, including cardiovascular and cortisol reactivity, inflammatory markers, mental health, quality of life, health behaviors, and cardiovascular risk factors, at 8 weeks and 6 months. We expect greater improvements in all secondary outcomes among HHT participants than in UC participants. This paper describes the study design, objectives and method.

## Method

### Design

**Figure 1:**
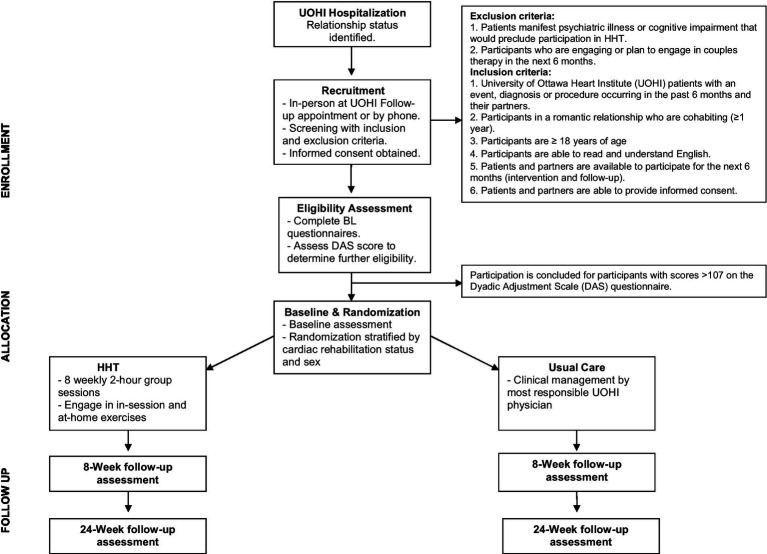
Study design and cohort flow.

The study is a single-centre, RCT with parallel group design. After a baseline assessment, participants are randomized in a 1:1 fashion, using a computer-generated sequence utilizing permuted blocks of 4, 8 and 10 initiated by the institutional methods centre, to the HHT program or to UC. Since sex and participation in CR can have an impact on the study outcomes, randomization is stratified by patient sex (male/female) and participation in the University of Ottawa Heart Institute (UOHI) CR program (yes/no). The trial began in October 2019 and data collection is ongoing. The protocol was approved by our institutional research ethics board (OHSN-REB# 20190101-01H) and all participants provide informed consent. Figure 1 depicts the study design and cohort flow.

### Participants

Patients at a large cardiac care hospital in Canada and their partners are approached to participate if they meet the study eligibility criteria, including: (1) being in a romantic relationship of any sexual orientation for at least 1 year; (2) living together for one or more years; (3) the patient having a cardiac event, procedure, or hospitalization in the past 6 months; (4) being 18 years or older; and, (5) being available to participate for 6 months. As a substantial number of participating couples reported high relationship satisfaction and, thereby, likely experience limited benefit from the HHT intervention, in July 2023, another inclusion criterion – one or both partners must score below the satisfied range on relationship quality (i.e., score of <107 on the Dyadic Adjustment Scale (DAS) questionnaire) (Spanier, 1976) - was added as advised by the study biostatistician. Exclusion criteria include: (1) those with any psychiatric or cognitive problems that would impair participation in the HHT program. Staff screen for impairment via chart review. If nothing is documented but a potential participant appears to have these impairments, the study psychologist would determine (either through further chart review or discussion with the research coordinator, referring clinician, or patient) whether participation is appropriate, or (2) planned or active participation in couples’ therapy. Since the HHT program materials were available in English only at the study outset, participants are required to read and understand English. However, the option to complete questionnaires in French or English or speak in their first language during some tasks is possible.

### Recruitment and data collection

Following their discharge, patients in a romantic relationship who provided institutional consent to be approached for research are directly contacted in-person or by phone by a research coordinator (RC). For those who do not provide such consent, a member of their circle of care initiates contact and obtains permission for the RC to contact the patient to provide information about the study. Recruitment strategies are adopted to achieve adequate participant enrolment, such as approaching patients at scheduled follow-up appointments and emailing additional information to those who indicate initial interest and confirm email consent. If patients express interest in participating following initial contact, the RC then explains the study procedures and obtains informed consent from the patient and partner. Participants are then invited to fill out an online questionnaire package via Research Electronic Data Capture (REDCap), a secure web-based application designed to support data capture for research studies. Those who prefer may complete the questionnaires on paper; these data are entered into REDCap by the RC. If DAS scores are above the established cut off, participation is concluded. Those in the eligible range are invited to complete the full study and scheduled for a baseline appointment at the UOHI.

#### Baseline assessment

Prior to the baseline assessment, participants fast, abstain from exercise for 12 h, and avoid caffeine and non-prescription diuretics for 4 h. At the assessment, they review and sign two copies of the study consent form. After obtaining signed consent, participants provide a blood sample (10 mL) and rinse their mouth in preparation for the saliva samples. Staff then attach pre-gelled electrodes under the participant’s right clavicle and below the left ribcage for HRV recordings. Saliva samples are collected 10 min after arrival. Then, participants complete the spousal disagreement form, followed by a 15-min rest period during which participants sit quietly but avoid sleeping. The conflict resolution task is completed next. Immediately before and after the conflict resolution task, blood pressure (BP) and heart rate readings are recorded, and additional saliva samples are taken. Participants are asked to rest for another 15 min upon completion of the second set of BP readings. Two final saliva samples are collected at 20 and 40 min after the conflict resolution task (Birkett, 2011). Finally, height, weight, waist circumference, and carbon monoxide levels are recorded, and patients are given an accelerometer to wear for a 7-day period. Bloodwork is collected by staff phlebotomist, and assessments are carried out by research staff with backgrounds in nursing, psychology, and health sciences. Study staff are trained on assessment procedures, including the reading of BP results and protocols for abnormal values or adverse events during the appointments. At the end of the baseline assessment, the RC randomizes participants to the HHT or UC and informs them of group assignment. Randomly generated intervention allocations are contained within sealed opaque envelopes. Research staff who assist with assessments are not present during randomization. Couples in the intervention arm receive information about HHT group logistics.

#### Follow-up assessments

The same procedures are conducted at each follow-up assessment (8 weeks and 6 months). A REDCap link is sent to participants via email or, if preferred, printed questionnaires are sent by post to be completed before the assessment. Patients also report if they enrolled in CR and, if so, in which program options they participated (e.g., exercise, nutrition, stress management class). They also indicate if they partook in any individual or couples-based psychological services. Research staff conducting follow up data collection, intervention infidelity checks, and behavioral coding are blind to the randomized condition. To maximize participant retention, assessments are scheduled to coincide with regular follow-up appointments or rehabilitation sessions at the UOHI whenever possible. Appointment reminders and parking passes are provided at each assessment, and gift cards are distributed after their final assessment. Efforts are made to collect primary outcome data despite potential deviation from study protocols. For example, the completion of online questionnaires will still be offered to those who might withdraw from in-person study activities.

#### Data management

Following study assessments, research assistants enter data into SPSS. Other staff members periodically carry out quality checks, and discrepancies are recorded and reported to the staff leading data entry. After assessment completion and data entry, files with participant information are stored in locked cabinets on site. To maintain confidentiality, all data entered is coded with study ID numbers instead of personal identifying information.

### Interventions

#### Healing hearts together intervention

Couples are invited to attend 8 weekly 2-h sessions facilitated by clinical psychologists with EFT training. Before providing the intervention, facilitators review information on CVD and its treatments, theory and practice of group interventions, and familiarize themselves with the HHT facilitator materials. An HHT facilitators manual was developed to standardize intervention delivery (Tulloch et al., 2017). Planned group size is 3–8 couples, with sessions available in the day or evening to accommodate participants’ work schedules. Since the onset of the COVID-19 pandemic (March 2020), however, groups have been conducted online using the secure platform Zoom; only one group was held in-person before the pandemic. During the program, participants are guided through seven conversations in which they learn to identify and improve communication patterns for positive interactions in relationships (Tulloch et al., 2021). EFT interventions allow couples to acknowledge their fears and vulnerabilities and solidify their sense of security and emotional connection by guiding partners to respond to these feelings in comforting ways (Johnson, 2004; Johnson, 2008). The intervention maintains principles from the *Hold Me Tight* program (Johnson, 2010) but, as alluded to above, centres on identifying the impact of CVD on relationship dynamics, including the recognition and processing of worries and emotions following a cardiac diagnosis or event, healing interactions, and discussing sexuality in a health context (Tulloch et al., 2021). Materials, such as in-class and take-home worksheets, didactic presentations, and videos, are incorporated to facilitate these conversations (Johnson, 2010; Tulloch et al., 2021). The session-by-session overview has been previously described (Tulloch et al., 2021). In brief, each session begins with a review of the previous session and its homework, followed by a didactic presentation, a video clip of a couple discussing EFT principles, a private dyadic conversation, a final group discussion, and a homework assignment. In addition, each couple receives a copy of the book, *Hold Me Tight*, in print or audio version (Johnson, 2008) to be read in-between sessions. Attendance is noted at each session. Participants are asked to inform research staff if they must miss a session, and if this is the case, they are sent presentation slides from the respective session for review. Participant feedback surveys are sent via email after each session. All sessions incorporate group discussions and private conversations between patients and partners. Group discussion allows couples to share experiences related to CVD with their partners and peers. Session two allows for participants to discuss their experiences as patients in a patient-only group and as partners of someone with CVD in a partner-only group discussion, both of which are guided by a facilitator. Zoom break-out rooms are used for dyadic-specific exercises; facilitators assist during these discussions. HHT intervention materials may be found here: https://iceeft.com/healing-hearts-together-relationship-education-program/.

#### Usual care

All patients at the study hospital are scheduled for a follow-up visit 4–8 weeks after hospitalization. At this appointment, a physician conducts a clinical assessment and determines appropriate medical management. Patients are automatically referred to the centre’s CR program, which includes exercise training and potential referrals to medical and allied health services, including dietetics, social work, psychology, and vocational rehabilitation; patients may choose whether they wish to receive these services or not. Optional attendance at nursing-led patient/caregiver support groups (e.g., heart failure support) and peer-support groups (e.g., Women at Heart) is also available. While the control group is potentially extensive, it represents evidence-based care for patients with CVD and, as such, preventing participants from enrollment would be unethical.

### Measures

Assessments are conducted at baseline before randomization, and at 8 weeks and 6 months; the timeline for all measures is presented in Table 1. As previous couples-based research often failed to include partners in their measurements, all participants complete measures assessing relationship variables, mental health, general Quality of Life (QoL), and health behaviors; are monitored for HRV, heart rate, and BP; and provide salivary and plasma samples. Disease-specific QoL, medication compliance, and CR adherence (if enrolled) is collected for patients only.

**Table 1:** Study measures and timeline.

Procedure/measure	Patient				Partner			
	Screen	BL	Week 8	Week 24	Screen	BL	Week 8	Week 24
Eligibility criteria	✓	✓			✓	✓		
Informed consent		✓				✓		
Demographics		✓				✓		
Medical history		✓				✓		
Follow-up medical questionnaire			✓	✓			✓	✓
Dyadic Adjustment Scale (DAS)		✓	✓	✓		✓	✓	✓
Traditional Masculinity-Femininity Scale (TMF)		✓				✓		
Salivary cortisol, HRV, and BP (pre-post a conflict-resolution task)		✓	✓	✓		✓	✓	✓
Brief Romantic Relationship Interaction Coding Scheme (BRRICS)		✓	✓	✓		✓	✓	✓
Experiences in Close Relationships Scale-12 (ECR-12)		✓	✓	✓		✓	✓	✓
Global Measure of Sexual Satisfaction (GMSEX)		✓	✓	✓		✓	✓	✓
Self-Efficacy in Romantic Relationships scale (SERR)		✓	✓	✓		✓	✓	✓
Beck Depression Inventory Second Edition (BDI-II)		✓	✓	✓		✓	✓	✓
Patient Health Questionnaire (PHQ-9)		✓	✓	✓		✓	✓	✓
General Anxiety Disorder 7 item (GAD-7)		✓	✓	✓		✓	✓	✓
Cardiac Anxiety Questionnaire (CAQ)		✓	✓	✓				
PTSD Checklist for DSM-5 (PCL-5)		✓	✓	✓		✓	✓	✓
Zarit Burden Interview (ZBI)						✓	✓	✓
Short Form 36 Health Survey (SF-36)		✓	✓	✓		✓	✓	✓
Heart-related Quality of Life Scale (HeartQoL)		✓	✓	✓				
Quality of Life of Cardiac Partners Questionnaire (QL-SP)						✓	✓	✓
Smoking status		✓	✓	✓		✓	✓	✓
Physical activity (ActiGraph GT3X-BT)		✓	✓	✓		✓	✓	✓
Cardiovascular markers (blood work)		✓	✓	✓		✓	✓	✓
Godin Leisure Time Exercise Questionnaire		✓	✓	✓		✓	✓	✓
Medication adherence		✓	✓	✓				
CR adherence		✓	✓	✓				
HHT end-of intervention measures (8 weeks)	Group Climate Questionnaire (GCQ) HHT Satisfaction Survey HHT intervention fidelity checklist							

#### Demographic and clinical information

Participants report their demographic (e.g., age, education, ethnicity) and clinical information (e.g., medical history, comorbidities, medications). Staff also extract patients’ medial history from their hospital chart. To explore how gender-related self-perceptions influence health outcomes and relational dynamics, participants complete the *Traditional Masculinity-Femininity (TMF) Scale,* a 6-item measure of self-ascribed masculinity and femininity. Psychometric properties have been established (Cronbach’s alpha = 0.94) (Kachel et al., 2016).

Relationship quality is assessed in 2 ways: (1) The primary outcome, participants’ perceived RQ, is evaluated using the *Dyadic adjustment scale (DAS)*, a 32-item questionnaire with well-established psychometric properties (e.g., Cronbach’s alpha = 0.96) (Spanier, 1976; Prouty et al., 2000; Spanier and Thompson, 1982). The DAS has been employed in research and clinical settings among various populations, including patients with heart disease (Spanier, 1976; Prouty et al., 2000; Spanier and Thompson, 1982). Scores range from 0 to 151, with higher scores indicating higher RQ. Scores ≥108 indicate high couple satisfaction; scores ≤70 indicate extreme couple distress (divorcing couples) (Spanier, 1976). The four subscales measure couple satisfaction, cohesion, consensus and affectionate expression (Spanier, 1976). (2) Couple dynamics are recorded during a 15-min conflict-resolution task, during which couples are instructed to discuss their top 3 topics that regularly cause disagreement in their relationship such as health, money, sex, or in-laws, with the intention of coming to a resolution. These recordings are then coded by 2 blinded independent reviewers (psychology staff and graduate students) using the *Brief Romantic Relationship Interaction Coding Scheme (BRRICS)* (Humbad et al., 2011), a standard procedural guideline for coding behaviors in romantic relationships. Routine spot checks are conducted by co-PI Greenman (blinded) to ensure reliability among the coders. The BRRICS evaluates both individual and dyadic aspects of interactions between partners, including positive and negative affect, positive and negative reciprocity, demand-withdraw patterns, and overall relationship satisfaction (Wildey and Burt, 2018).

Other relationship-related variables include attachment style, sexual satisfaction, and relationship self-efficacy. Participants complete the *Experiences in Close Relationships Scale-12 (ECR-12)* (Lafontaine et al., 2015) assessing attachment orientations using the dimensions of attachment anxiety and attachment avoidance with two subscales of six items each (rating from strongly disagree = 1 to strongly agree = 7). Low scores on both subscales indicate higher levels of secure attachment (Lafontaine et al., 2015; Wei et al., 2007; Brennan et al., 1998). Validity and reliability (Cronbach’s alpha = 0.74–87) of the scale has been established (Lafontaine et al., 2015). The adapted version of the *Global Measure of Sexual Satisfaction (GMSEX),* a 6-item measure, was used to assess overall satisfaction with the sexual relationship. Each item is rated on a 7-point bipolar scale (e.g., good-bad, valuable-worthless), with total scores ranging from 6 to 42, where higher scores indicate greater satisfaction (Lawrance and Byers, 1998). The psychometric performance of the scale is strong (e.g., Cronbach’s alpha = 0.96) (Quinn-Nilas, 2023). The *Self-Efficacy in Romantic Relationships scale (SERR)* is a 12-item questionnaire measuring confidence in romantic relationships. Participants indicate on a 9-point scale (1 = do not agree at all; 9 = completely agree) how much they agree with statements such as “I am just one of those people who is not good at being a romantic relationship partner” or “I feel insecure about my ability to be a good romantic partner.” (Riggio et al., 2013) Scores are totaled in a positive direction (i.e., higher scores indicate higher self-efficacy in a romantic partnership). The measure has been deemed valid and reliable (e.g., Cronbach alphas >0.79) (Riggio et al., 2013).

#### Mental health

Participants symptoms of depression over the past week are measured with the 21-item *Beck Depression Inventory Second Edition (BDI-II)* (Beck et al., 1996). Total scores range from 0 to 63; <13 = minimal, 14–19 = mild, 20–28 = moderate, and ≥29 = severe. The BDI-II demonstrated strong high internal consistency (Cronbach’s alpha = 0.90–0.93) and test–retest reliability (García-Batista et al., 2018). The *Patient Health Questionnaire (PHQ-9)*, a 9-item, 4-point Likert scale (0 = not at all; 3 = nearly every day) assessing depressive symptoms over the past 2 weeks is also completed by participants. Scores range from 0 to 27; cut-off scores for moderate, moderately severe and severe depressive symptoms are 10, 15, and 20, respectively (Kroenke and Spitzer, 2002). The tool shows strong psychometric properties, including good internal consistency (Cronbach’s alpha = 0.89) and robust validity (Kroenke et al., 2001). Two measures of depression were used because they provide complementary information. The PHQ-9 coincides with DSM-IV criteria for major depressive disorder. The BDI-II is more detailed measure that assesses the severity of cognitive, behavioral, and physiological symptoms of depression. Participants’ generalized anxiety is evaluated with the *General Anxiety Disorder 7 item (GAD-7)* questionnaire, a 4-point Likert scale with a total score of 0–21, where 10–14 and 15–21 indicate moderate and severe anxiety, respectively (Spitzer et al., 2006). The GAD-7 demonstrated strong reliability, with a Cronbach’s alpha of 0.89 and a composite reliability index of 0.90. It also exhibited robust validity across procedural, criterion, construct, and factorial measures (Bolgeo et al., 2023). Patient participants’ cardiac specific anxiety was assessed with the 18-item *Cardiac Anxiety Questionnaire (CAQ).* The *CAQ* yields 3 subscales reflecting fear, heart-focused attention, and related avoidance. Patients rate, on a 5-point Likert scale (0 = never, 4 = always), how frequent a behavior occurs (e.g., “I avoid activities that make my heartbeat faster”). Responses are summed for a total score; scores >26 are considered elevated (Eifert et al., 2000). The CAQ has shown good internal consistency (Cronbach’s alpha = 0.083) and construct validity (Eifert et al., 2000; Leissner et al., 2022).

The *PTSD Checklist for DSM-5 (PCL-5)* is a 20-item questionnaire used to assess post-traumatic stress symptoms. Responses to trauma-related statements are rated on a 5-point scale (0 = not at all, 4 = extremely); scores ≥33 are indicative of probable PTSD diagnosis (Weathers et al., 2013). Participants are directed to focus on the cardiac event when responding. The PCL-5 demonstrates strong reliability and validity in assessing PTSD symptoms across diverse populations (Cronbach’s alpha>0.91) (Blevins et al., 2015). One partner-specific questionnaire, the 22-item *Zarit Burden Interview (ZBI),* assesses how partners feel when taking care of another person and indicates the degree of caregiver burden. Responses are indicated on a 5-point Likert scale (0 = never; 4 = nearly always). Total scores ≥17 are considered high burden (Al-Rawashdeh et al., 2016). Psychometric properties of the ZBI have been established (e.g., Cronbach alphas>0.80) (Bachner and O’Rourke, 2007). As noted, all the mental health scales have been validated and used with patients with cardiovascular disease (Al-Rawashdeh et al., 2016; Van Beek et al., 2012; Seng et al., 2010; Blanchard et al., 1996; Hammash et al., 2013; Moullec et al., 2015; Löwe et al., 2008).

#### Quality of life

General and disease-specific QoL is assessed with 3 questionnaires. All participants complete the *Rand 36-item Short Form Health Survey* (Ware et al., 1993) to assess QoL across eight domains: physical functioning, role limitations due to physical health, role limitations due to emotional problems, energy/fatigue, emotional well-being, social functioning, pain, and general health. The SF-36 is widely used and validated across various patient populations, including those with cardiovascular disease (Brazier et al., 1992; Ware and Gandek, 1998). Patients also complete the *Heart-related Quality of Life Scale (HeartQoL)* (Oldridge et al., 2014), a 14-item validated questionnaire designed to evaluate how heart disease impacts daily functioning. The HeartQoL includes a global health-related QoL score and separate physical and emotional subscales (Oldridge et al., 2014). Similarly, partners complete the *Quality of Life of Cardiac Spouses Questionnaire (QL-SP),* a validated 26-item measure with two subscales: emotional functioning (14 items) and physical and social functional dimensions (12 items) (Ebbesen et al., 1990). Psychometric properties have been established for all scales with Cronbach alphas exceeding 0.70 (Brazier et al., 1992; Oldridge et al., 2014; Thomson et al., 2013; Zangger et al., 2018).

#### Health behaviors

Smoking status is reported by participants and confirmed with CO concentration (<10 ppm) in an expired breath sample, using the Russell standard (West et al., 2005). Participants wear an *ActiGraph GT3X-BT accelerometer* on their non-dominant hand for 7 days to assess physical activity and sedentary behaviors. Self-reported frequency and duration of mild, moderate and vigorous physical activity is also measured using a modified and validated version of the *Godin Leisure Time Exercise Questionnaire (GLTEQ)* (Godin and Shephard, 1985). Two *adherence* measures are used: (1) Medication adherence is measured with 3-items (e.g., how often do you forget to take your medication?) and (2) CR adherence is calculated by the percentage of exercise sessions attended relative to those prescribed.

#### Physical/cardiovascular markers

Blood samples taken by a staff phlebotomist are analyzed for *total cholesterol (TC), high-density lipoprotein cholesterol (HDL-C), low-density lipoprotein cholesterol (LDL-C), triglycerides (TG), C-reactive protein (CRP), and hemoglobin A1C (A1C).* Saliva samples are collected using Salimetrics Oral Swab (Salimetrics, State College, PA) to assess *cortisol and inflammatory marker IL-6*. Following the Trier Social Stress Test Protocol (Birkett, 2011), 5 samples are taken: upon arrival, before and after the conflict resolution task and 20 and 40 min later. Samples are stored at −80°C and shipped frozen to Salimetrics for analysis. *Heart Rate* Var*iability (HRV)* is measured by continuous heart period recordings of R-R intervals sampled at the rate of 1,000 Hz using First Bodyguard 2 (Firstbeat Technologies, OyJyvaskyla, Finland) connected to 2 leads by pre-gelled (Ag/AgCl) disposable electrocardiograph electrodes attached beneath the right clavicle and left ribcage (Firstbeat Technologies, 2025). *BP* is measured with the BpTru using standardized procedures (Myers et al., 2008). Finally, weight, height and waist circumference are recorded.

#### HHT intervention participant evaluation

HHT group participants complete the *Group Climate Questionnaire (GCQ)* (MacKenzie, 1983), a 12-item instrument designed to assess group engagement, conflict and avoidance. Responses are recorded on a 7-point Likert scale, from 0 (not at all) to 7 (extremely). The treatment specific *HHT Satisfaction Survey*, a 22-item survey assessing participants’ experience and satisfaction with the HHT sessions and the online delivery of the intervention, is given to HHT participants after program completion.

#### HHT intervention attendance and fidelity

Participation in the HHT intervention is recorded by class *attendance* (% attended). To ensure that the HHT intervention is delivered as intended, two sessions of every 8-session group are randomly pre-selected, recorded and scored on study-specific *HHT intervention fidelity checklist* by two independent raters (psychology trainees). Points are given, and percentage calculated, for each key point that is outlined in the session-by-session HHT manual (Tulloch et al., 2017).

### Data monitoring

Adverse events are recorded and reported to the principal investigator for review. Endorsement of the suicide item of the PHQ-9 or BDI are reported immediately to one of the study psychologists for risk assessment and care. The Healing Hearts Together study was initially granted ethics approval without a Data Safety Monitoring Board (DSMB); it was not recommended nor inquired by ethics. Nonetheless, the PIs convened a DSMB committee in 2024, consisting of a psychiatrist, cardiologist and consulting statistician, to discuss data management for the study.

### Auditing

The study has undergone 2 routine internal audits: one divisional, investigator-initiated audit in February 2022 and an institutionally led audit in February 2023. No major infractions were noted.

### Statistical analyses

#### Sample-size calculation

An *a priori* calculation was performed using RQ as measured by the DAS at 8 weeks as the primary outcome. Sample size was derived in 3 steps. First, we examined the standard deviation (SD) of the DAS scores pre-post intervention (SDchange) of pilot patients and partners separately (i.e., HHT pilot data); all SDchange values were close to 10. The consensus of the study investigators and other clinical content experts was to identify a minimum clinically important difference (MCID) based on a small (0.20) to moderate (0.50) Cohen’s effect size. The difference between the HHT and UC groups in the mean change was 3.9 for a small to moderate effect size. This value is close to the commonly used minimum mean change score for the DAS (3.16) (Jacobson and Truax, 1991). Then, we used these values (SDchange = 10; MCID = 3.9) to calculate the sample size with PASS15 sample size software. With 210 couples assigned in a 1:1 fashion to the two groups, we will have 80% power to detect a difference of 3.9 in the change in the DAS between the HHT and UC groups (*α* = 0.05). Nonetheless, we aim to inflate our sample size to account for the design effect of clustering (design effect = 1 + (m-1)*ICC = 1.21 = 254 couples), assuming that our average group size (m) will be 8 couples and our ICC is 0.030. We further increased our sample size target to 304 couples to account for an expected 20% loss to follow-up. As such, we aim to recruit 304 couples but will have sufficient power to detect differences in the primary outcome with 210 couples.

#### Planned statistical analyses

SPSS and HLM statistical software will be used to analyze the study data. Demographic and clinical variables reported by participants who complete assessments will be compared to those lost to follow-up to verify the representativeness of the cohort. Analyses will be with the intent to treat sample. Research staff record all cases of missing data, including non-retention of participants or withdrawal of consent. Where data are missing, the frequency and reasons for missing data will be recorded. Missing data will be statistically examined for patterns of systematic missingness, and an appropriate missing data strategy will be employed. In situations where data are missing, the data collected up to that point will be used, provided the participant has not withdrawn their consent or requested that the information be removed. For the *primary outcome*, a multilevel analysis of covariance with individuals’ DAS values at 8 weeks as the dependent variable and DAS baseline scores as the covariate at level 1 of the model. The study conditions (HHT vs. UC) to which couples were assigned will be dummy-coded and entered at level 2. To evaluate *secondary outcomes,* a three-level multi-level model for repeated measurements will be utilized. The analysis will include repeated measurements within individuals in Level 1, between-individual variability in Level 2, and couples in Level 3. Within-couple slope variances will be fixed at Level 2 but allowed to vary at Level 3 (Atkins, 2005). A linear parameter will model changes in outcomes from baseline to 8 weeks to 24 weeks. The study condition (HHT vs. UC) will be dummy-coded and modeled as a fixed effect at Level 3. Baseline patient control variables will be entered at Level 2. Binary outcomes will be modeled utilizing a logit link. Improved model fit between nested models will be evaluated using deviance statistics and a chi-square distribution. Effect sizes will be assessed with the pseudo-R squared statistic or Cohen’s d when appropriate. Full maximum likelihood estimation method will be applied for all analyses. For both primary and secondary analyses, we will also conduct stratified analyses for all outcomes by sex (male/female), CR participation (yes/no), and relationship quality (distressed/not distressed on the DAS).

### Anticipated outcomes

It is expected that participants randomized to HHT intervention will report higher RQ (≥3.9 points on the DAS) and improved secondary outcomes as compared to those in UC.

### Dissemination plans

Trial results will be shared through published manuscripts to participants, healthcare professionals, and the public. To enhance the accessibility of the research findings, results will also be disseminated in interactive formats. For example, findings will be presented during hospital rounds and in-services to inform clinical staff of the importance of relationship quality and encourage the inclusion of the partner in the care team. Results will be disseminated to research participants and the lay public in formats such as community presentations, in-person research dissemination events, and media releases.

## Discussion

To our knowledge, the HHT randomized trial is one of few studies evaluating a relationship-enhancement intervention specifically designed for couples facing the challenges of CVD. The HHT intervention is rooted in an evidence-based theoretical framework, expert recommendations (Johnson, 2004; Johnson, 2008), and direct input from couples managing CVD (Tulloch et al., 2020). Further, the intervention and trial design address many methodological limitations in the couples-based literature in the cardiac domain, including small sample sizes; samples restricted to specific cardiac populations limiting generalizability; data collection limited to patients only; limited and solely subjective measures of RQ, and, few relevant and cardiac-specific health outcomes. Indeed, the current trial has several strengths, including the RCT design, comprehensive assessments using validated, objective and self-report measures, partner-specific data collection, and aims to recruit a large sample. Our proof-of-concept data showed pre-post improvements in RQ, mental health and quality of life (Tulloch et al., 2021). The present RCT now tests if the HHT intervention is better than usual care on many relationship and health outcomes. With significant findings, the finalized intervention will signal if effectiveness testing is required and may indicate that the intervention should be systematically implemented at other cardiac care sites, as well as pave the way for future intervention research for other chronic conditions (e.g., cancer). If positive, this research may also contribute to a shift from medical and physical indicators alone to a more holistic approach which comprises the promotion of enhanced social support through improved couple relationships, thereby enhancing cardiovascular outcomes. Considering that partners often share similar age and lifestyle factors as patients, they too may be vulnerable to poor mental or physical health outcomes, and programs such as HHT enhancing RQ may improve partners’ health along with improving the CVD recovery process for patients (Bouchard et al., 2019).

## Data Availability

The datasets presented in this article are not readily available because the study is ongoing. Requests to access the datasets should be directed to Heather Tulloch, hetulloch@ottawaheart.ca.
